# Artificial Intelligence-Based Histopathology Segmentation for Resource-Constrained Healthcare Systems

**DOI:** 10.3390/diagnostics16142146

**Published:** 2026-07-08

**Authors:** Tahir Mahmood, Su Jin Im, Muhammad Zubair, Kang Ryoung Park

**Affiliations:** 1 Division of Electronics and Electrical Engineering, Dongguk University, 30 Pildong-ro 1-gil, Jung-gu, Seoul 04620, Republic of Korea; tahirmahmood@dongguk.edu (T.M.); sujin125917@dgu.ac.kr (S.J.I.); 2 School of Computer Science, University of Galway, H91 TK33 Galway, Ireland; engr.zubairfarooqi@gmail.com

**Keywords:** histopathology segmentation, colorectal cancer, deep learning, resource-efficient neural network, gland segmentation, multi-scale feature extraction

## Abstract

**Background/Objectives:** Colorectal cancer (CRC) is one of the leading causes of cancer-related mortality worldwide, and accurate histopathological tissue segmentation is critical for timely and reliable diagnosis. Healthcare systems represent complex adaptive environments where diagnostic tools must function reliably across heterogeneous clinical settings, varying staining protocols, and resource-constrained infrastructures. However, existing deep learning segmentation models often require substantial computational resources, limiting their deployment in such settings. This study proposes a novel, resource-efficient colorectal histopathology segmentation network (RCHS-Net) designed for robust clinical deployment across diverse and resource-constrained healthcare environments. **Methods:** RCHS-Net employs a compact multi-scale encoder with channel recalibration blocks, a gland context module (GCM) with three parallel atrous convolutions and lightweight self-attention for multi-scale contextual feature extraction, and a feature pyramid decoder (FPD) for fine-grained spatial reconstruction. To address the demands of real-world healthcare systems, feature-wise linear modulation (FiLM) conditioning enables class-aware segmentation across multiple tissue categories, while MixStyle augmentation improves stain domain generalization across heterogeneous laboratory and scanner conditions. **Results:** The model was evaluated on two publicly available benchmark datasets: the EBHI-Seg dataset and the GlaS dataset. On EBHI-Seg, RCHS-Net achieved a mean Dice coefficient of 95.20% and a mean IoU of 91.10% across six colorectal tissue classes, with only 243,226 trainable parameters. On the GlaS benchmark, RCHS-Net attained a Dice score of 93.39% and an IoU of 88.32%, outperforming state-of-the-art methods. **Conclusions:** RCHS-Net demonstrates that high-accuracy histopathology segmentation can be achieved with a compact architecture, offering a scalable and practical solution for AI-assisted cancer diagnosis across the complex, heterogeneous conditions of real-world healthcare systems, supporting scalable and equitable cancer diagnostics globally.

## 1. Introduction

Colorectal cancer (CRC) stands as one of the most serious and widespread forms of malignancy. In terms of cancer incidence, it ranks fourth among men and third among women worldwide, underscoring the critical importance of early and precise diagnosis for successful therapeutic intervention [1,2]. The microscopic analysis of tissue specimens stained with hematoxylin and Eosin (H&E) continues to serve as the most reliable and widely accepted method for diagnosing colorectal and kidney cancers, equipping pathologists with comprehensive insights into structural tissue patterns, cellular arrangement, and the degree of tumor differentiation [3,4]. However, manual examination of histological slides is inherently time-consuming, labor-intensive, and subject to inter-observer variability, particularly in high-volume clinical settings and resource-constrained environments. The digitization of pathology slides into whole-slide images (WSIs) has opened new avenues for computational methods to assist pathologists [5].

As CRC population screening programs continue to expand across countries worldwide, the number of resected biopsy specimens requiring evaluation, particularly polyps, has risen considerably. Artificial intelligence offers a promising solution for reducing the demanding workload associated with the classification and documentation of these samples. Among these computational approaches, deep learning-based image segmentation has emerged as a particularly powerful tool, enabling pixel-level delineation of tissue regions and tumor boundaries that are critical for clinical decision-making. Convolutional neural networks (CNNs), and particularly encoder–decoder architectures such as U-Net [6], have demonstrated strong performance in medical image segmentation tasks. The field of medical image segmentation has undergone considerable transformation owing to rapid progress in deep learning ability to extract multi-level feature representations from complex data structures [7]. Unlike conventional approaches that struggle with complex medical imaging data, deep learning architectures, most notably convolutional neural networks (CNNs), demonstrate a remarkable. More recently, hybrid architectures combining CNNs with attention mechanisms and vision transformers have further improved segmentation accuracy [8]. Among the most impactful developments in medical image processing are vision transformers, which leverage self-attention mechanisms to simultaneously model short- and long-range spatial dependencies within an image. This capacity to integrate both local and global contextual information enables vision transformers to achieve superior segmentation performance compared to CNNs, which are inherently constrained by their local receptive fields. However, such models typically demand substantial computational resources and memory, raising significant barriers for deployment in low-resource clinical settings. These computational demands are particularly problematic in the context of complex healthcare systems, where diagnostic AI must function reliably across heterogeneous infrastructures, varying staining protocols, and diverse clinical environments.

Notwithstanding the remarkable progress achieved in this field, a substantial disconnect persists between the robust performance of deep learning models and their real-world clinical implementation. Training architectures such as CNNs, fully convolutional networks, and attention-based models necessitate considerable computational resources, particularly high-performance graphics processing units (GPUs), which pose significant obstacles in low-resource settings where conventional techniques remain more practically feasible owing to their reduced computational requirements. This issue is especially pronounced in developing nations and point-of-care environments, where access to advanced hardware infrastructure is scarce, yet the demand for reliable and precise cancer detection is at its highest [9,10]. Consequently, there is a pressing need for lightweight yet accurate segmentation architectures that can be deployed on edge devices without significant loss of diagnostic performance. Furthermore, existing benchmark evaluations report results on a single dataset, limiting the generalization of proposed methods across diverse tissue types and imaging conditions. Addressing these challenges requires approaches grounded in complex systems thinking, models that are not only accurate but also resource-aware, domain-adaptive, and deployable across the full spectrum of real-world healthcare settings.

To address this gap, we propose a novel resource-efficient colorectal histopathology segmentation network (RCHS-Net) for class-conditioned binary segmentation in resource-constrained clinical environments. The proposed RCHS-Net employs a compact multi-scale encoder based on ShuffleNet V2 with squeeze-and-excitation (SE) channel recalibration blocks, which adaptively emphasize informative feature channels while suppressing less relevant responses, thereby improving discriminative feature representation with minimal computational overhead. A gland context module (GCM) incorporating dual parallel atrous convolutions and lightweight self-attention captures contextual information over multiple spatial scales, enabling the network to model both fine glandular structures and larger gland clusters without the computational expense of transformer- or pooling-based context aggregation. A feature pyramid decoder (FPD) progressively fuses multi-scale encoder representations to recover fine spatial details and accurately delineate gland boundaries. Feature-wise linear modulation (FiLM) conditioning enables tissue-specific feature adaptation across the six EBHI Seg tissue categories with minimal additional parameters, allowing a single lightweight model to accommodate substantial variations in gland morphology between tissue types. MixStyle augmentation is incorporated at the early encoder stages to improve robustness to inter-laboratory staining variations, one of the primary sources of domain shift in digital pathology, without increasing the model complexity or inference cost. The proposed framework is evaluated on the publicly available EBHI-Seg and GlaS benchmarks, demonstrating state-of-the-art performance for both multi-tissue class-conditioned gland segmentation and binary gland segmentation while maintaining high computational efficiency.

The main contributions of this work are as follows:

- We propose RCHS-Net, a compact encoder–decoder architecture for class-conditioned binary segmentation with only 243,226 trainable parameters, designed specifically for deployment in resource-constrained clinical environments and edge devices. The architecture employs channel recalibration blocks that selectively emphasize diagnostically relevant H&E channel responses, achieving strong feature discrimination within a sub-250K parameter footprint, smaller than any comparable architecture in the literature.
- We introduce a novel GCM combining three parallel atrous convolutions at complementary dilation rates (2, 6, and 12) with a lightweight spatial self-attention mechanism. The three branches capture crypt-level, gland-level, and gland cluster-level structures, while the self-attention addresses incomplete gland delineation at patch boundaries, achieving multi-scale contextual understanding without the overhead of full transformer-based attention.
- We propose a class-conditioned stain-aware learning strategy integrating FiLM conditioning and MixStyle augmentation. FiLM enables a single compact model to adapt to the distinct glandular architectures of all six tissue classes, while MixStyle simulates inter-laboratory H&E stain variation to improve robustness across different scanners and pathology laboratories. This strategy directly addresses the generalization demands of complex, heterogeneous healthcare environments.
- Extensive experiments on two public benchmarks (EBHI-Seg and GlaS) confirm state-of-the-art performance on both datasets while maintaining a sub-250K parameter footprint. To support reproducibility and community adoption, the source code is publicly available on GitHub https://github.com/tahirlee/RCHS-Net-segmentation-in-histopathology-images (accessed on 18 November 2025) [11].

## 2. Related Work

Over the past decade, the domain of colorectal histopathology image analysis has witnessed substantial progress. The related work can be broadly categorized into four groups: handcrafted feature-based methods, convolutional neural network-based deep learning methods, attention and transformer-based methods, and lightweight resource-efficient architectures.

### 2.1. Handcrafted Feature-Based Methods

Early computational approaches to colorectal histopathology analysis relied heavily on manually engineered features extracted from H&E-stained tissue images. Traditional machine learning methods depend on a prior feature extraction stage, relying on handcrafted feature vectors to learn discriminant functions; both shallow and ensemble methods require explicitly designed representations. Common strategies included texture descriptors such as gray-level co-occurrence matrices (GLCM), local binary patterns (LBP), and Gabor filters, which were combined with classifiers such as support vector machines (SVMs) and random forests to distinguish between benign and malignant tissue regions. Among the foundational contributions in this area, the work of Kather et al. [12] stands out as one of the most extensively referenced, introducing a collection of 5000 histological images spanning eight distinct tissue categories derived from human colorectal cancer specimens and systematically assessing a broad set of texture descriptors and classification approaches. The most effective classification strategy identified in their study demonstrated a notable improvement in tumor-stroma separation, with accuracy advancing from 96.9% to 98.6%, surpassing the performance of previously established methods.

Shi et al. [13] evaluated five classical machine learning segmentation methods on the six-class EBHI-Seg benchmark: k-means clustering, Markov random field (MRF), OTSU thresholding, the watershed algorithm, and Sobel edge detection. Their results demonstrated that k-means achieved the best overall performance with a maximum Dice score of approximately 0.65, while OTSU and watershed consistently underperformed due to difficulties separating foreground from background in complex tissue images. While handcrafted methods offered interpretability and low computational cost, they struggled with high inter-class similarity, intra-class variability, and staining protocol differences, fundamental limitations that motivated the shift toward end-to-end deep learning approaches.

### 2.2. CNN-Based Deep Learning Methods

The emergence of convolutional neural networks (CNNs) marked a transformative shift in histopathology image segmentation. Unlike handcrafted approaches, CNNs learn hierarchical representations directly from data, capturing subtle nuances in tissue morphology, including color variations, texture differences, and spatial relationships, yielding superior segmentation accuracy and consistency. Encoder–decoder architectures, particularly U-Net and its variants, have become the de facto standard for pixel-level tissue segmentation due to their ability to combine high-level semantic features with fine-grained spatial detail through skip connections. Shi et al. [13] also evaluated three deep learning methods (SegNet, U-Net, and MedT) on the EBHI-Seg dataset, finding that SegNet and U-Net achieved average evaluation indices of approximately 0.90, substantially outperforming all classical machine learning methods, while MedT achieved metrics between 0.70 and 0.80 at the cost of a significantly larger model size and longer training time. Isensee et al. [14] proposed nnU-Net, a self-adaptive segmentation framework capable of autonomously adjusting its preprocessing pipelines, architectural configurations, and training procedures in accordance with the characteristics of a given dataset, thereby setting a robust benchmark that has consistently outperformed competing approaches across a wide range of medical image segmentation competitions.

In the context of polyp segmentation within colonoscopy images, Fan et al. [15] developed PraNet, a model that utilizes parallel reverse attention to consolidate features across multiple scales, enabling precise delineation of polyp boundaries. Building upon this line of research, Dong et al. [16] presented Polyp-PVT, which adopts a pyramid vision transformer as its backbone encoder, complemented by a cascaded fusion module and a camouflage identification module, collectively designed to mitigate noise interference and enhance the richness of extracted features for improved polyp segmentation. Addressing the challenge of gland structures that vary significantly in size, shape, and appearance, Yuan and Cheng [17] proposed a U-Net-based multi-scale context fusion algorithm that extracts rich contextual information at different encoding stages and assigns adaptive weights to multi-scale semantic features through a top-down bidirectional skip-feature fusion module. The key problem tackled is that gland structures vary significantly in size, shape, and appearance, while staining variability and complex tissue morphology make segmentation difficult. Liu et al. [18] proposed residual pyramid attention U-Nett++ (RPAU-Net++), integrating ResNet-50 residual feature extraction, a joint pyramid fusion module with dense dilated convolutions at four dilation rates, and a convolutional block attention module into the U-Net++ framework, significantly outperforming mainstream segmentation models in both IoU and Dice metrics.

### 2.3. Vision Transformer-Based Methods

The integration of attention mechanisms and vision transformers into medical image segmentation has advanced performance in histopathology analysis by enabling models to capture long-range spatial dependencies beyond the receptive field of standard convolutions. Recent studies have extended deep learning in medical imaging through efficient localization networks for ultrasound imaging and transformer-based multiple instance learning for computational pathology, highlighting the growing importance of contextual feature modeling across different medical imaging tasks [19,20]. TransUNet [21] pioneered the combination of vision transformer encoders with U-Net-style decoders, demonstrating that transformer self-attention effectively models global context; the updated formulation supporting both 2D and 3D implementations achieved significant Dice score improvements over nnU-Net on pancreatic tumor and multi-organ segmentation tasks. SwinCup [22] incorporates a hierarchical Swin Transformer architecture with shifted window attention as its encoding component, enabling efficient extraction of both global and multi-scale contextual representations, which are subsequently processed through a cascaded upsampling decoder for refined segmentation output. Cao et al. [23] presented MT-SCnet, a transformer-based network designed for microscopic image segmentation that integrates multi-scale token decomposition with spatial-channel attention fusion. The architecture partitions image tokens into non-overlapping sub-regions while simultaneously combining spatial and channel attention mechanisms to strengthen feature representations across multiple resolutions. A hybrid ResUNet–EfficientNet–Swin transformer framework [24] for colorectal cancer histopathology, it leverages residual learning, efficient feature extraction, and self-attention jointly, outperforming conventional CNN-Transformer hybrids that typically combine only a single CNN backbone with a transformer. The swin transformer within this framework captures long-range dependencies through shifted-window multi-head self-attention with relative positional bias, making it effective for high-resolution histopathology images.

Despite these advances, transformer-based architectures carry significant computational burdens. The multi-head self-attention mechanism exhibits quadratic complexity with respect to sequence length, making full transformer models memory-intensive and slow to train on high-resolution histopathology tiles. This computational bottleneck severely limits their applicability in resource-constrained clinical settings, motivating the development of lightweight alternatives.

### 2.4. Lightweight and Resource-Efficient Architectures

The necessity of deploying deep learning models within clinically constrained settings and on edge computing devices has spurred increasing research interest in the development of segmentation architectures that are both lightweight in design and efficient in terms of parameter utilization. Lightweight neural networks provide smaller memory footprints, accelerated inference speeds, and reduced energy consumption compared to large-scale counterparts, making them well-suited for edge computing scenarios where high-end GPU resources are unavailable.

In the domain of colorectal histopathology, Barua et al. [25] conducted the first investigation integrating MobileViT as an encoder within the U-Net framework for six-class tissue segmentation on the EBHI-Seg dataset, comparing it against VGG16-U-Net, ResNet50U-Net, and MobileNeU-Net backbones across binary cross-entropy and Dice loss configurations. In a parallel effort targeting cross-tissue generalization, a multi-scale attention and channel-wise feature fusion network (MAC-Net) [26] integrating multi-scale feature fusion, channel-wise attention, and a spatial-pooling bottleneck was trained on EBHI-Seg and cross-validated on GlaS, demonstrating the importance of enriched encoder–decoder lateral connections for accurate gland segmentation across diverse tumor differentiation stages. A lightweight U-Net variant inspired by ConvNeXt (LUNeXt) [27] employs depthwise separable convolutions to capture global features with fewer parameters, achieving reduced parameter counts and floating-point operations compared to standard U-Net while maintaining competitive accuracy across multiple medical image segmentation benchmarks.

Despite the progress reviewed above, a critical gap persists in the literature: no existing work has demonstrated a sub-250K-parameter model capable of accurate multi-class colorectal histopathology segmentation across all six EBHI-Seg tissue categories while simultaneously incorporating mechanisms for stain-domain generalization and class-conditioned feature adaptation. The proposed RCHS-Net directly targets this gap by combining a compact multi-scale encoder, a novel GCM, Feature-wise linear modulation (FiLM), and MixStyle augmentation within a unified architecture explicitly designed for clinical deployment in resource-constrained environments. Table 1 summarizes the strengths and limitations of representative methods reviewed above, contextualizing the design decisions of the proposed RCHS-Net.

## 3. Materials and Methods

### 3.1. Overview of the Proposed Methodology

**Figure 1:**
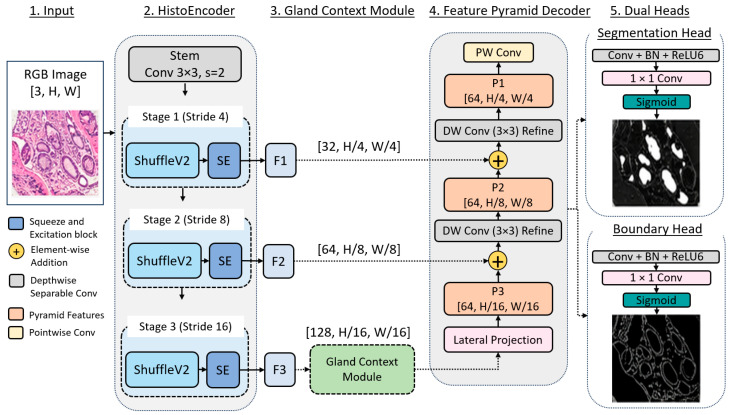
Overview of the proposed method.

The proposed method, RCHS Net, is a lightweight encoder–decoder network for class-conditioned binary histopathology segmentation, designed for deployment in resource-constrained healthcare environments. As illustrated in Figure 1, the architecture comprises five components: a multi-scale HistoEncoder with SE recalibration, a gland context module with dual parallel atrous convolutions, lightweight self-attention at the bottleneck, a feature pyramid decoder for top-down spatial recovery, a segmentation head for pixel-wise binary tissue classification, and an auxiliary boundary head for training-time gland-wall supervision. FiLM conditioning enables class-aware feature adaptation across all six tissue categories, while MixStyle augmentation ensures stain domain generalization. The complete architecture maintains a sub-250K parameter footprint, making it suitable for edge-device deployment. The proposed RCHS-Net performs class-conditioned binary segmentation rather than conventional multi-class semantic segmentation. This is an intentional design choice that enables a single lightweight network to segment multiple tissue categories through FiLM-based feature modulation while sharing a common backbone, thereby maintaining a compact model suitable for resource-constrained healthcare environments. Each component is described in detail in the following subsections.

### 3.2. Detailed Architecture of the Proposed RCHS-Net

#### 3.2.1. RCHS-Net Encoder (HistoEncoder)

The encoder of RCHS Net is a compact three-scale feature-extraction backbone, termed HistoEncoder, designed to capture multi-scale histopathological structures through progressively downsampled feature representations. It is built using lightweight ShuffleNet V2 [28] blocks augmented with SE [29] channel recalibration, enabling a strong hierarchical representation within a constrained parameter budget. ShuffleNet V2 provides efficient accuracy to a computation trade-off, where channel shuffling enhances cross-channel interaction and improves feature diversity without increasing parameters. The progressive channel expansion from 24 to 32 to 64 to 128 aligns with increasing feature complexity across scales, allowing deeper stages to encode richer contextual information while avoiding redundancy at initial stages. SE blocks further improve robustness by adaptively reweighting channel responses, suppressing stain-dependent variations, and emphasizing structurally relevant features. In addition, stride-2 convolutions are used instead of pooling to preserve spatial detail during downsampling, ensuring that the fine spatial information required for accurate boundary delineation is retained for the decoder.

The complete encoder produces a tuple ${(F}_{1},\;F_{2},\;F_{3})$ passed to the decoder’s lateral connections and the GCM, respectively, as in Equation (1):

(1) $$HistoEncoder\left(X\right)=\;F_{1},\;F_{2},\;F_{3}$$

The encoder begins with a stem module that halves the spatial resolution and projects the three-channel RGB input into an initial 24-channel representation as in Equation (2):

(2) $$X_{stem}=\;{ReLU6(BN(conv}_{3\times 3,\;s=2}(X)))\;\epsilon \;R^{B\times 24\times \frac{H}{2}\times \frac{W}{2}}$$

In the stem module, a stride-2 convolution is preferred over a pooling layer because it preserves learnable spatial structure from the very first layer, which is critical when fine gland boundaries must later be recovered at full resolution.

Each stage is built from ShuffleNet V2 blocks, which avoids the expensive element-wise addition of residual networks by instead operating on split and shuffled channel groups. For a stride-1 block, the input tensor x of C is split into two equal halves $\left(x_{a},\;x_{b}\right)$ along the channel dimension. The right branch applies a depthwise-separable transform as represented by Equation (3):

(3) $$x_{b}^{\prime }=\;PW(DW\left(PW\left(x_{b}\right)\right))$$

where PW(.) denotes a 1 ×1 pointwise convolution followed by batch normalization (BN) and ReLU6, and DW(.) denotes a depthwise 3 ×3 convolution followed by BN. The block output is obtained by concatenation and channel shuffling as shown by Equation (4):

(4) $$ShuffleV2(x)=Shuffle(Concat(x_{a},\;x_{b}^{\prime }))$$

For a stride-2 block, both branches downsample the full input and concatenate their outputs, effectively doubling the channel count while halving spatial resolution without any pooling operation. Channel shuffling promotes cross-group gradient flow and inter-channel feature mixing at negligible computational cost, which is an important property for a sub-megaparameter model where every parameter must contribute to multiple feature channels. After this, three consecutive stages of ShuffleV2 blocks process the stem features, producing multi-scale feature maps at strides 4, 8, and 16 relative to the input as represented by Equations (5)–(7):

(5) $$F_{1}=\;{SE}_{1}\left(S_{1}\left(X_{stem}\right)\right)\;\epsilon \;R^{B\times 32\times \frac{H}{4}\times \frac{W}{4}}$$

(6) $$F_{2}={SE}_{2}\left(S_{2}\left(F_{1}\right)\right)\;\epsilon \;R^{B\times 64\times \frac{H}{8}\times \frac{W}{8}}$$

(7) $$F_{3}={SE}_{3}\left(S_{3}\left(F_{2}\right)\right)\;\epsilon \;R^{B\times 128\times \frac{H}{16}\times \frac{W}{16}}$$

where $S_{k}(.)$ denotes the k-th stage comprising one stride-2 ShuffleV2 block followed by three (stage 1) or four (stages 2 and 3) stride-1 blocks, and ${SE}_{k}(.)$ is the SE recalibration applied after each stage. A SE block is applied after each stage to learn a set of channel-wise importance weights that selectively emphasize diagnostically relevant feature maps, specifically, those encoding hematoxylin-stained nuclear chromatin and Eosin-stained cytoplasmic structures while suppressing less informative channels. For a stage output $F\;\epsilon \;R^{B\times C\times H^{\prime }\times W^{\prime }\;}$, the SE block computes as shown by the Equations (8)–(10):

(8) $$z=GAP\left(F\right)\;\epsilon \;R^{B\times C}$$

(9) $$s=\sigma (W_{2}ƍW_{1}z))\;\epsilon \;R^{B\times C}$$

(10) $$SE\;\left(F\right)=F\;ʘ\;s\;[:,\;:,\;None,\;None\}$$

where $GAP\left(.\right)$ denotes global average pooling over the spatial dimensions, $W_{1}\;\epsilon \;R^{\frac{C}{r}\times C}$ and $W_{2}\;\epsilon \;R^{C\times \frac{C}{r}}$ are learned linear projections with a reduction ratio r=4, δ(⋅) is ReLU, σ(⋅) is the sigmoid function, and ⊙ denotes channel-wise multiplication broadcast across spatial dimensions. The bottleneck dimension ⌊C/r⌋ is clamped to a minimum of 16 to prevent information collapse in shallow-channel stages.

In this HistoEncoder, all convolutional weights are initialized with Kaiming normal initialization calibrated to the ReLU nonlinearity, and all BatchNorm scale and shift parameters are initialized to one and zero, respectively. This ensures stable gradient magnitudes at the start of training, even for the deepest stage, which otherwise suffers from compounded initialization variance across the stacked depthwise-separable layers. Table 2 presents the layer-wise details of the HistoEncoder of the proposed method.

#### 3.2.2. GCM

The GCM serves as a semantic bottleneck bridge between the encoder and the decoder. It receives the deepest encoder feature map $F_{3}\;\epsilon \;R^{B\times 128\times \frac{H}{16}\times \frac{W}{16}}$ and enriches it with multi-scale contextual information before passing the refined representation to the FPD. The module is specifically designed to address one of the fundamental challenges in gland segmentation: glands appear at highly variable sizes within a single tissue section, ranging from small, isolated crypts to large multi-gland clusters, and a single convolutional receptive field is insufficient to capture this diversity simultaneously. In this module, before the multi-scale branches, a pointwise convolution compresses the 128-channel input to half of its capacity, reducing the memory consumption and computational cost of the parallel branches without sacrificing representational diversity, as shown by Equation (11):

(11) $$H_{o}=\;PW(F_{3})\;\epsilon \;R^{B\times 64\times \frac{H}{16}\times \frac{W}{16}}$$

where PW(.) denotes a 1 ×1 pointwise convolution followed by BN and ReLU6. After this, two parallel atrous (dilated) depthwise-separable convolutions then process the compressed feature map $H_{o}$ simultaneously, each with a different dilation rate as represented by Equation (12):

(12) $$B_{r}=\;{DW}_{r}\left(H_{o}\right),\;r\;\epsilon \;\{2,6\}$$

where ${DW}_{r}$(.) denotes a depthwise-separable convolution with a dilation rate r, followed by BN and ReLU6. Each branch captures a distinct scale of spatial context without any increase in the number of parameters relative to a standard convolution, since dilation expands the effective receptive field purely by spacing the kernel sampling positions. The two dilation rates correspond to two clinically meaningful contextual ranges in histopathological tissue. The branch with r=2 captures fine intra-glandular detail, including the transition between the gland lumen and the surrounding epithelial wall, which is critical for precise boundary delineation. The branch with r=6 encodes context at the scale of a complete individual gland unit, allowing the module to reason about the full extent of a single gland in one receptive field. The feature maps produced by the two branches are combined through concatenation along the channel dimension and subsequently unified to restore the complete 128-channel width by means of a convolution operation, as can be shown by Equation (13):

(13) $$H_{1}=PW\left(Concant\left(B_{2},\;B_{6}\right)\right)\;\epsilon \;R^{B\times 128\times \frac{H}{16}\times \frac{W}{16}}$$

Following multi-scale fusion, a lightweight self-attention mechanism is applied to $H_{1}$ to model long-range spatial dependencies across the feature map. Standard multi-head self-attention is prohibitively expensive at this stage due to the quadratic complexity with respect to the number of spatial tokens. The proposed module instead uses a single-head formulation with a reduced key dimensionality of $d_{k}=24$, which reduces the projection cost by 4× relative to full-channel keys while preserving the ability to capture global spatial correlations. Query, key, and value projections are computed using 1×1 convolutions as shown by Equation (14):

(14) $$Q=\;W_{Q}H_{1},\;K=\;W_{K}H_{1},\;V=\;W_{V}H_{1}$$

where $W_{Q}$, $W_{K}\;\epsilon \;R^{d_{k}\times C}$ and $W_{V}\;\epsilon \;R^{C\times C}$ with *C* = 128, and $d_{k}=24$. The spatial dimensions are flattened to obtain $N=\frac{H}{16}\cdot \frac{W}{16}$ tokens, and scaled dot-product attention is computed as in Equations (15) and (16):

(15) $$A=\;Softmax\;\left(\frac{Q^{T}K}{\sqrt{d_{k}}}\right)\;\epsilon \;R^{N\times N}$$

(16) $$H_{attn}=\left(V\;.\;A^{T}\;\right)\;\epsilon \;R^{B\times C\times \frac{H}{16}\times \frac{W}{16}}\;$$

The attended output is projected back to the channel space through a 1×1 convolution followed by BN and added as a residual to the attention input inside the module, ensuring that the self-attention refines rather than replaces the fused multi-scale features. After this, a final projection with a skip connection from the original encoder input $F_{3}$ produces the context-enriched output as in Equation (17):

(17) $$F_{ctx}=ReLU\left(W_{proj}H_{attn}+\;F_{3}\right)\;\epsilon \;R^{B\times 128\times \frac{H}{16}\times \frac{W}{16}}$$

The residual connection to $F_{3}$ is a critical design choice. It ensures that the original encoder gradient path is preserved throughout training. If the GCM produces a near-zero contextual refinement early in training, the gradient still flows cleanly through the skip term to the encoder weights. This prevents the module from acting as a gradient bottleneck during the initial training epochs when the atrous branches and attention weights are not yet calibrated.

The GCM addresses limitations that are particularly acute in gland segmentation, and that cannot be resolved by the encoder or decoder alone. The encoder, operating with stride-2 downsampling at each stage, has a limited effective receptive field at the finest scales where boundary detail must be preserved. By the time features reach $F_{3}$ at stride 16, a single 3×3 convolution covers only a 3×3 pixel neighborhood in the original image space. The self-attention component complements the atrous branches by enabling truly global reasoning. While atrous convolutions expand the receptive field geometrically, they are still localized in the sense that each output position attends only to a fixed spatial neighborhood. Self-attention, by contrast, allows any two spatial positions to directly exchange information regardless of their distance, which is important for resolving ambiguous regions where isolated tissue fragments could be mistaken for gland lumina. By keeping the key dimension at $d_{k}=24$ rather than the full channel width, the attention mechanism focuses on a compressed semantic subspace that captures the most discriminative structural patterns (gland shape, lumen topology, and epithelial arrangement) while remaining insensitive to low-level texture variation from staining differences.

**Figure 2:**
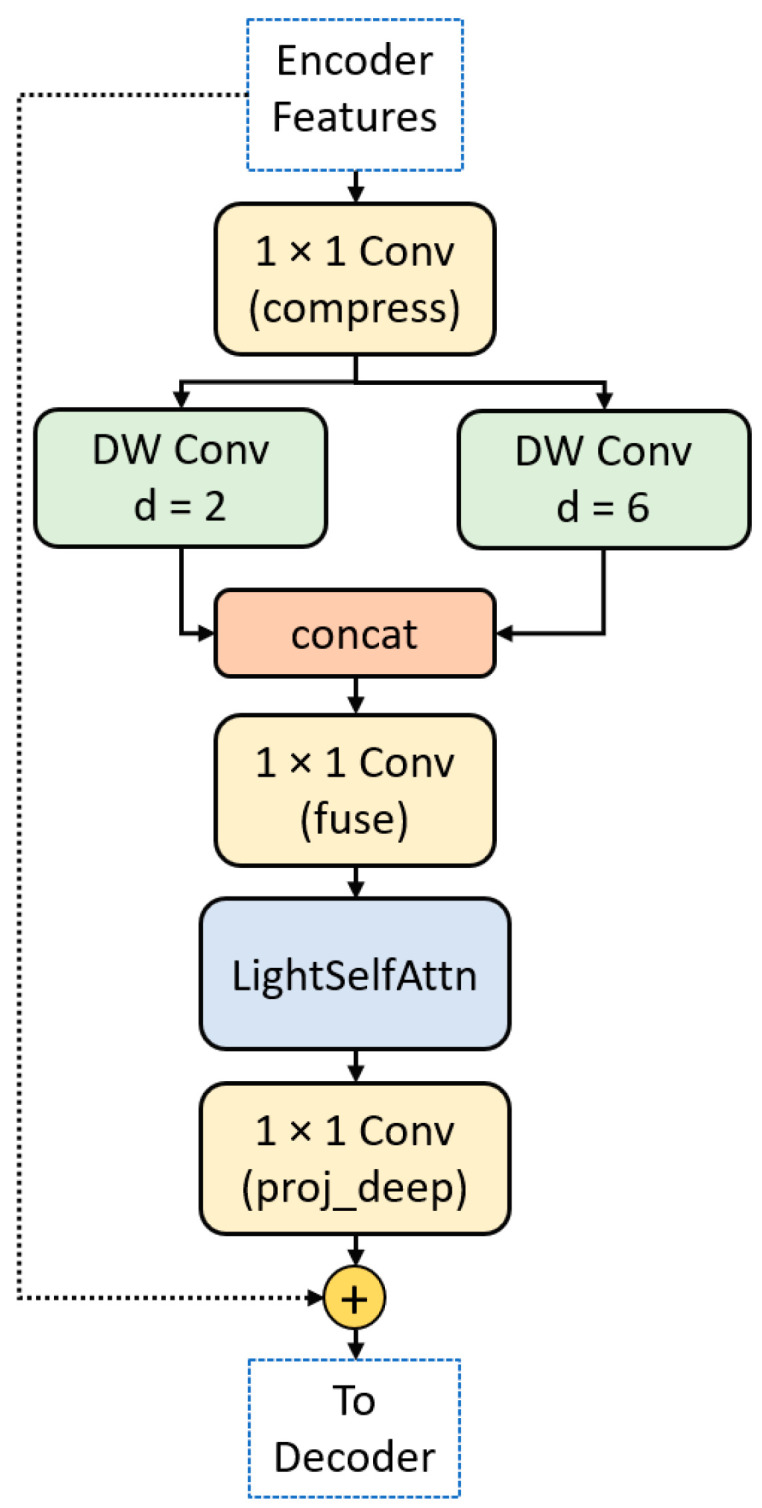
Overview of the GCM module of the proposed method. Solid arrows indicate the primary feature propagation path, while the dashed arrow represents a skip connection.

**Figure 3:**
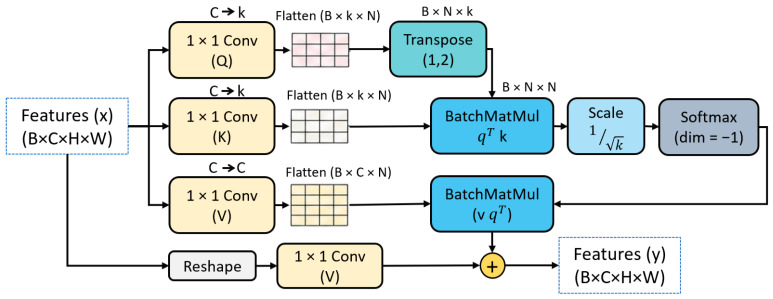
Lightweight self-attention mechanism of the GCM module.

Together, the dual atrous fusion and lightweight self-attention make the GCM the primary source of semantic understanding in RCHS-Net, producing a context-enriched representation $F_{\mathrm{ctx}}$ that encodes not just what tissue structures are present locally, but how they relate spatially to their neighborhood across the full range of glandular scales present in H&E-stained colorectal tissue. Figure 2 and Figure 3 show the overview diagram and self-attention mechanism of the GCM module, while Table 3 presents the layer-wise details of the GCM module of the proposed method.

#### 3.2.3. FPD

The FPD is the spatial recovery component of RCHS-Net. Its role is to progressively reconstruct the full-resolution segmentation map from the context-enriched bottleneck representation $F_{\mathrm{ctx}}$, reintroducing fine-grained spatial detail lost during the encoder’s progressive downsampling by fusing encoder skip connections at each matching resolution level. The decoder follows a strict top-down design: semantic context flows downward from the coarsest scale, and spatial detail flows laterally inward from the encoder at each level, ensuring that every pyramid level is informed by both high-level glandular semantics and precise boundary information.

A fundamental challenge in multi-scale feature fusion is that encoder and decoder features at the same resolution do not share the same channel count, making element-wise addition impossible without first projecting them into a common channel dimension. The FPD resolves this with lightweight 1×1 pointwise lateral projection layers that map all feature maps (from both the encoder and the GCM) to a uniform decoder width of 64 channels, as shown in Equation (18):

(18) $$P_{3}={Lat}_{3}\left(F_{ctx}\right)\;\epsilon \;R^{B\times 64\times \frac{H}{16}\times \frac{W}{16}}$$

where ${\mathrm{Lat}}_{3}(\cdot )$ denotes a 1×1 convolution followed by BN and ReLU6 that projects from 128 channels to 64 channels. Similarly, lateral projections for the encoder skip features are defined as in Equation (19):

(19) $${Lat}_{2}\left(F_{2}\right)=\;\epsilon \;R^{B\times 64\times \frac{H}{8}\times \frac{W}{8}},\;{Lat}_{1}(F_{1})=\;\epsilon \;R^{B\times 64\times \frac{H}{4}\times \frac{W}{4}}$$

By performing all projections before fusion rather than after, the FPD ensures that no spatial information is mixed until both feature streams are already in a common representational space, preserving the full discriminative content of each encoder scale.

Starting from $P_{3}$ at the coarsest resolution, the decoder builds progressively finer representations through a two-step operation at each level: bilinear upsampling to match the target resolution, followed by element-wise addition with the corresponding lateral encoder feature, and then a depthwise-separable refinement convolution to consolidate the merged representations as represented by Equations (20) and (21):

(20) $$P_{2}=\;{Ref}_{2}\left(Up\left(P_{3}\right)+\;{Lat}_{2}\left(F_{2}\right)\right)\;\epsilon \;R^{B\times 64\times \frac{H}{8}\times \frac{W}{8}}$$

(21) $$P_{1}={Ref}_{1}\left(Up\left(P_{2}\right)+{Lat}_{1}\left(F_{1}\right)\right)\;\epsilon \;R^{B\times 64\times \frac{H}{4}\times \frac{W}{4}}$$

where Up(⋅) denotes bilinear interpolation to the spatial dimensions of the target encoder feature map, and ${\mathrm{Ref}}_{k}(\cdot )$ is a 3×3 depthwise-separable convolution followed by BN and ReLU6. A final 1×1 pointwise projection produces the unified decoder feature map as in Equation (22):

(22) $$F_{\mathrm{ctx}}=PW(P_{1})\;\epsilon \;R^{B\times 64\times \frac{H}{4}\times \frac{W}{4}}$$

The feature map $F_{\mathrm{ctx}}$ at stride 4 retains sufficient spatial resolution to resolve individual gland boundaries while carrying the semantic context propagated from the bottleneck, making it the ideal input for both the segmentation and boundary prediction heads.

The FPD makes two specific contributions to histopathology segmentation quality. First, the strict maintenance of a fixed 64-channel width throughout the decoder (rather than progressively increasing channel count at finer scales as in many dense prediction decoders) means that maintaining a fixed 64-channel width throughout the decoder provides a consistent feature capacity at every pyramid level, enabling intermediate representations at H/8 and H/4 to retain semantic information while keeping the decoder computationally efficient. Second, by anchoring the top of the pyramid to $F_{\mathrm{ctx}}$ from the GCM rather than directly to $F_{3}$ from the encoder, the decoder inherits the multi-scale atrous context and self-attention refinement performed by the GCM at every subsequent pyramid level through the top-down propagation path. Every spatial position in $P_{2}$ and $P_{1}$, therefore, implicitly encodes information about the multi-gland context that surrounds it, even though neither of these levels directly applies atrous convolutions or attention. This property is critical for resolving ambiguous regions where isolated tissue fragments must be distinguished from gland lumina by their spatial relationship to surrounding structures rather than their local appearance alone.

The feature map D serves as the shared representation for both the segmentation and boundary prediction heads. Unlike conventional multi-class semantic segmentation networks that simultaneously predict all tissue categories, the proposed framework performs class-conditioned binary segmentation. Specifically, the segmentation head predicts the binary gland mask corresponding to the tissue category provided as auxiliary input, while FiLM conditioning adapts the shared feature representation according to the specified tissue class. During inference, the tissue category is assumed to be available as part of the routine clinical workflow through biopsy request information, specimen metadata, or pathology records, and is used solely to condition the feature modulation process rather than being predicted by the segmentation network itself. This design enables a single compact model to share parameters across all tissue categories while preserving category-specific feature adaptation [30,31]. Table 4 presents the layer-wise details of the FPD block, while Table 5 summarizes the prediction heads and FiLM conditioning module.

## 4. Results

### 4.1. Experimental Datasets

Two publicly available histopathology benchmark datasets are used to evaluate the proposed RCHS-Net: the Early Barrett’s Histopathology Image Segmentation (EBHI-Seg) [13] dataset for colorectal histopathology image segmentation, and the Gland Segmentation (GlaS) [32] dataset for gland segmentation in colorectal adenocarcinoma. Together, these datasets provide complementary evaluation scenarios. EBHI-Seg evaluates generalization across six distinct pathological grades on a larger and more diverse image collection, while the GlaS dataset challenges the model with high inter-patient stain and tissue variability on a small, curated set. Given that both datasets are publicly available for academic use in accordance with the terms stipulated by their respective providers, the requirement for informed consent was waived for the purposes of this study. The following subsection provides the details about the datasets.

#### 4.1.1. EBHI-Seg

The EBHI-Seg [13] dataset represents a large-scale, publicly accessible benchmark specifically curated for colorectal histopathology segmentation tasks, encompassing a total of 4456 images, which include 2228 H&E-stained histopathological section images alongside their corresponding 2228 pixel-level ground truth annotation masks. The dataset was collected by two histopathologists at the Cancer Hospital of China Medical University and annotated by 12 biomedical researchers following a consistent labeling protocol. All sections were obtained through intestinal biopsy at a magnification of 400× (10× eyepiece, 40× objective) using a Nikon Olympus microscope. Images are provided at a fixed resolution of 224 × 224 pixels. The dataset spans six pathological categories reflecting the progression from normal colonic tissue to invasive malignancy: Normal (76 images), Polyp (474 images), Low-grade Intraepithelial Neoplasia (639 images), High-grade Intraepithelial Neoplasia (186 images), Serrated Adenoma (58 images), and Adenocarcinoma (795 images). Table 6 summarizes the class distribution of this dataset.

**Figure 4:**
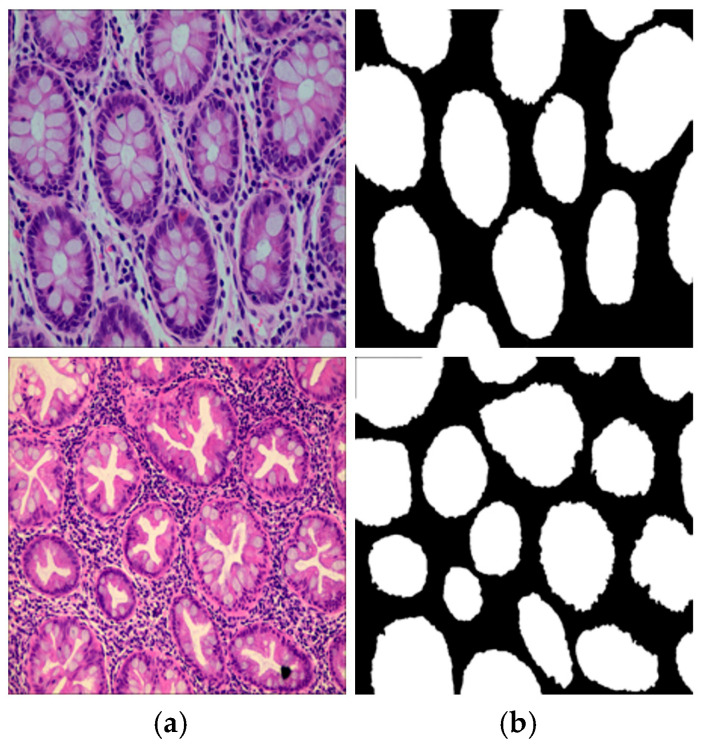
Sample images of the EBHI-Seg dataset. (**a**) represents the input frame and (**b**) represents the corresponding ground truth mask, respectively.

For a fair comparison with previous studies, we adopted the same training, validation, and test split as reported in the [13], presented in Table 7. The validation set was completely disjoint from the test set and was used exclusively for early stopping and model selection, while the final performance was evaluated on the independent test set. The number of images in each subset for all tissue categories is summarized in Table 7, while Figure 4 presents the sample images from this dataset.

#### 4.1.2. GlaS

The GlaS [32] dataset was introduced as part of the MICCAI 2015 Gland Segmentation Challenge and remains the primary benchmark for evaluating automated gland segmentation methods in colorectal histopathology. The dataset encompasses 165 images sourced from 16 H&E-stained histological sections representing stage T3 or T4 colorectal adenocarcinoma, with each section corresponding to a unique patient and processed through independent laboratory procedures. Such a construction deliberately fosters considerable inter-subject diversity with respect to both staining characteristics and the organizational patterns of tissue, rendering the dataset a particularly stringent benchmark for evaluating the capacity of models to generalize across varying domain conditions.

The histological sections were digitally scanned into whole-slide images (WSIs) using a Zeiss MIRAX MIDI Slide Scanner (Carl Zeiss MicroImaging GmbH, Jena, Germany) at a pixel resolution of 0.465 µm and rescaled to 20× objective magnification. A total of 52 visual fields were extracted from both malignant and benign tissue regions across all WSIs, selected to encompass the widest possible diversity of tissue architectural patterns. An experienced pathologist graded each visual field as benign or malignant based on overall glandular architecture and manually annotated the boundaries of individual glandular objects within each field, with these annotations serving as the segmentation ground truth. The visual fields were subsequently subdivided into smaller non-overlapping patches, each inheriting the grade of its parent visual field, yielding a final dataset of 85 training and 80 test images. The original dataset splits provided by the creators were retained to ensure fair accuracy comparisons.

**Figure 5:**
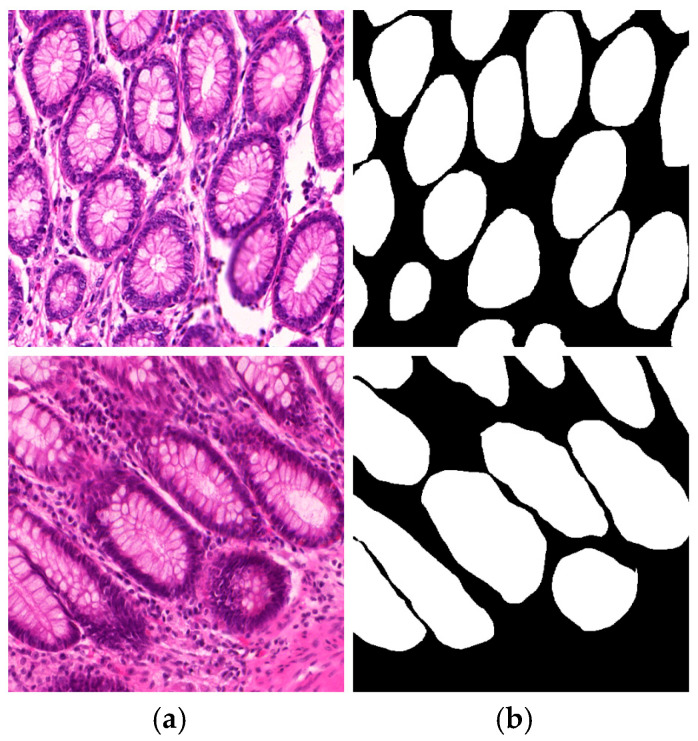
Sample images of the GlaS dataset. (**a**) represents the input frame and (**b**) represents the corresponding ground truth mask, respectively.

For a fair comparison with previous studies, we retained the original training and test split provided by the GlaS challenge organizers [32]. Since the official benchmark does not define a separate validation set, 10% of the training images were randomly selected as a validation set for model selection and early stopping, while the remaining training images were used for network optimization. The validation set was completely disjoint from the official test set, and all reported performance was obtained on the held-out test set. Sample images from this dataset are presented in Figure 5.

### 4.2. Data Augmentation

Histopathology images exhibit substantial variability in stain intensity, tissue orientation, and illumination conditions across different laboratories, slides, and acquisition systems. To improve the generalization capability of RCHS-Net and reduce overfitting on the relatively small GlaS training set, a multi-level data augmentation pipeline is applied during training. Augmentations are applied on-the-fly to each training batch and are never applied during validation or testing.

The augmentation pipeline is organized into three categories. Geometric augmentations include random horizontal and vertical flipping, random rotation up to 90°, and random cropping with resizing back to the target resolution of 224 × 224 pixels. These operations are applied identically to both the input image and its ground truth mask to maintain spatial correspondence. Photometric augmentations include random adjustments to brightness, contrast, saturation, and hue within bounded ranges, as well as random Gaussian blurring, simulating the variability in staining intensity and focus quality commonly encountered across different pathology laboratories. These color-space perturbations are applied to the image only and not to the ground truth mask.

In addition to standard augmentations, MixStyle is incorporated as a domain-level stain augmentation strategy. MixStyle interpolates the channel-wise mean and variance statistics of feature maps from two randomly paired training samples within a mini-batch, effectively synthesizing new stain appearances that the network has not encountered during training. This is applied at the output of the first two encoder stages, where the feature statistics are most closely correlated with low-level stain appearance rather than high-level tissue structure. MixStyle operates during training only and introduces no additional parameters or inference overhead. By exposing the encoder to a continuous distribution of interpolated stain styles, MixStyle improves the robustness of the learned representations to the inter-laboratory stain variation that is the dominant source of domain shift in digital pathology benchmarks.

In the proposed method, no stain normalization or stain color standardization was applied. Instead, the proposed framework relies on photometric augmentations together with MixStyle-based feature level augmentation to improve robustness to staining variability across different laboratories. In addition, no patch extraction was performed, as both the EBHI Seg and GlaS datasets provide pre extracted image patches. All images were resized to 224 × 224 pixels prior to training and evaluation.

### 4.3. Experimental Setup and Training

All experiments are conducted on a workstation equipped with an Intel Core i7-7700 CPU operating at 3.60 GHz, 24 GB of system RAM, and an NVIDIA GeForce GTX 1070 GPU [33] with 8 GB of dedicated video memory. The relatively modest hardware configuration motivated the lightweight design of the proposed RCHS-Net, which operates well within this memory budget at the training batch size and resolution used. The proposed method is implemented using the PyTorch deep learning framework (version 1.8.1). All experiments are conducted under a fixed random seed to ensure reproducibility of data splits, weight initialisation, and augmentation sequences.

The proposed RCHS-Net model was trained from scratch using a hybrid Dice and Binary Cross-Entropy loss function together with the AdamW optimizer. The two loss terms are combined with equal (1:1) weighting, total = L_dice + L_bce, without additional tuning, since the dataset is approximately balanced at the pixel level and therefore does not require compensatory class weighting. Boundary target masks are generated from the binary ground-truth mask via morphological erosion with a 3 × 3 elliptical kernel. MixStyle is applied only during training, at the first two encoder stages (32- and 64-channel features), with probability *p* = 0.5 per batch. For the GlaS benchmark, which provides no tissue-category annotation, FiLM conditioning is not applied; the GlaS variant of RCHS-Net uses the same encoder–GCM–FPD backbone without the FiLM/class-embedding pathway, since there is no class signal to condition on.

The training process used an initial learning rate of 0.0001, a weight decay of $1\times 10^{-4}$, and a batch size of 16, while training was performed for a maximum of 150 epochs. A cosine annealing learning rate scheduling strategy with a 5 epoch warmup stage was adopted to improve optimization stability and convergence behavior during the early training stages. Gradient clipping with a threshold value of 0.5 was additionally applied to prevent unstable parameter updates during backpropagation. The input images were resized to 224×224 pixels, and light augmentation was applied during training to improve the generalization capability of the model. An early stopping strategy with a patience value of 10 epochs was employed to prevent unnecessary optimization and terminate training when no meaningful improvement was observed in the validation performance.

**Figure 6:**
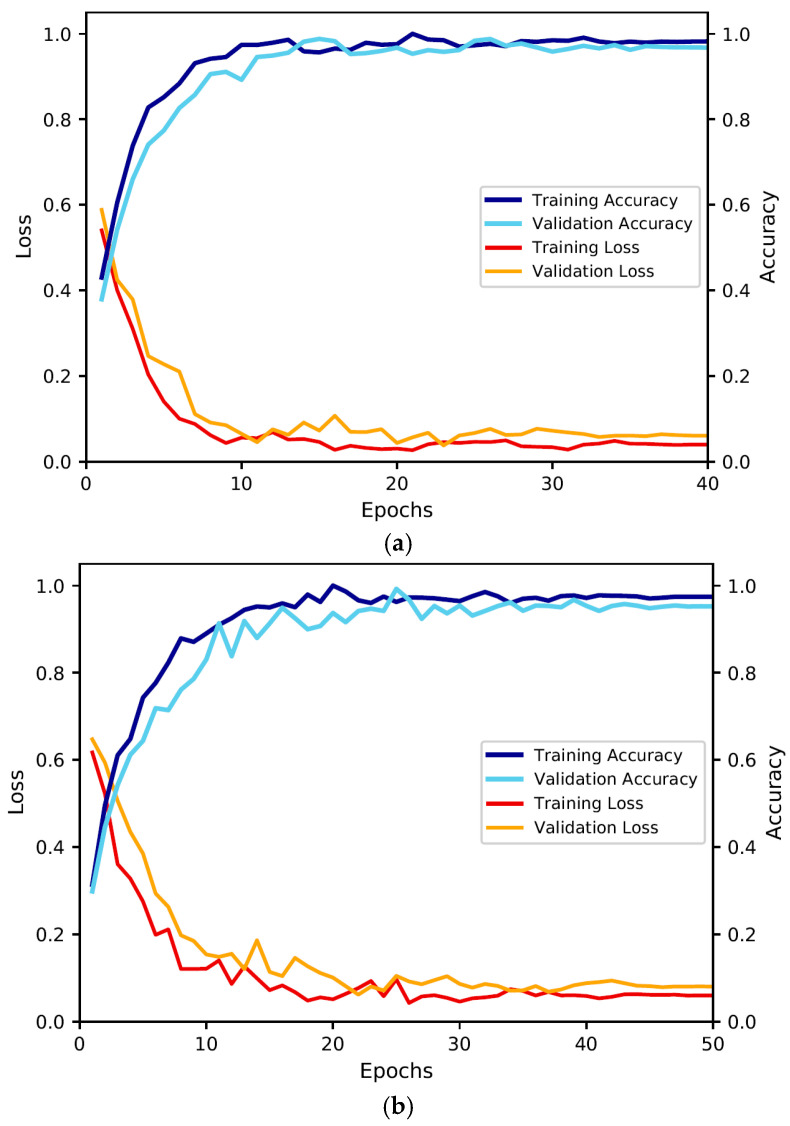
Training and validation accuracy and loss curves of the proposed RCHS Net on the two benchmark datasets. (**a**) presents the curves for the EBHI Seg dataset, while (**b**) shows the corresponding curves for the GlaS dataset.

The performance of the model during training and validation was assessed through examination of the corresponding accuracy and loss curves, as depicted in Figure 6. The progressive convergence of these curves confirms that the proposed model successfully learned meaningful representations from the input data. While minor fluctuations were noted in the early epochs, attributed to stochastic optimization and data augmentation procedures, both curves gradually stabilized and converged as training advanced. The close alignment between training and validation accuracy throughout the process indicates that the model generalized well to previously unseen data without signs of overfitting. Likewise, the parallel convergence of the training and validation loss curves affirms stable optimization and the absence of underfitting. Taken together, these findings highlight the robustness, stability, and overall effectiveness of the proposed RCHS-Net framework in addressing histopathological image segmentation tasks.

### 4.4. Evaluation Metrics

The proposed method’s performance was assessed using five standard segmentation metrics: Dice coefficient, Jaccard Index (IoU), Conformity coefficient, Precision, and Recall. The Dice coefficient measures the overlap between predicted and ground-truth segmentation masks, and is defined as 2|P∩G| / (|P|+|G|), where P and G denote the predicted and ground-truth regions, respectively. The Jaccard Index, also known as Intersection over Union (IoU), is related to Dice but penalizes false positives and false negatives more strongly, computed as |P∩G| / |P∪G|. The conformity coefficient (CC) was computed to quantify the agreement between the predicted and ground truth segmentation masks. A higher conformity coefficient indicates better segmentation performance, with a value of 1 corresponding to perfect agreement. Precision measures the proportion of predicted positive pixels that are truly positive, while Recall measures the proportion of true positive pixels that are correctly identified. Together, these five metrics provide a comprehensive evaluation of both region-level overlap accuracy and boundary-level segmentation quality.

### 4.5. Ablation Study

To evaluate the individual contribution of each architectural component in RCHS-Net, we conduct a systematic component-wise ablation study on the EBHI-Seg dataset. Starting from a minimal base encoder–decoder (HistoEncoder + FPD without any auxiliary modules), we progressively add the GCM, the SE channel recalibration FiLM class conditioning, the auxiliary boundary head, and MixStyle stain augmentation. All variants share identical training hyperparameters, data splits, and random seeds to ensure a fair comparison. Results are reported in terms of mean Dice coefficient and mean IoU across the six EBHI-Seg tissue categories.

The results in Table 8 reveal several key findings. The base HistoEncoder–FPD configuration (Variant A) achieves a mean Dice of 90.83% and IoU of 83.47%, establishing a competitive starting point given the compactness of the backbone. Adding SE channel recalibration (Variant B) yields a +1.31% Dice improvement, confirming that channel-wise reweighting of hematoxylin and Eosin responses provides meaningful discriminative benefit at minimal parameter cost (+10K parameters). The introduction of the GCM (Variant C) provides the largest single-component gain, raising mean Dice to 93.68% (+1.54% over B) and IoU to 88.07%, demonstrating that multi-scale atrous context and lightweight self-attention are the most impactful architectural contributions for capturing the range of glandular structures present in colorectal histopathology. FiLM class conditioning (Variant D) adds a further +0.73% Dice and +1.43% IoU over Variant C, confirming that explicit per-class feature modulation is necessary for a single compact model to handle the heterogeneous glandular morphologies of all six EBHI-Seg tissue categories. The auxiliary boundary head (Variant E) contributes an additional +0.45% Dice and +0.72% IoU through sharpened gland-wall supervision during training, with no inference overhead since the boundary head is discarded at deployment. Finally, MixStyle stain augmentation (full RCHS-Net, Variant F) improves mean Dice by +0.34% and IoU by +0.88% over Variant E, demonstrating the value of explicit stain domain regularization for robustness to inter-laboratory H&E variation. Taken together, the ablation study confirms that each component of RCHS-Net makes a distinct and complementary contribution to the final segmentation performance, and that removing any single component results in a measurable degradation. The GCM is the single most impactful module, while SE recalibration, FiLM conditioning, boundary supervision, and MixStyle augmentation each provide consistent incremental improvements that collectively raise the mean Dice by 4.37 percentage points above the minimal base configuration while keeping the total parameter count within the sub-250K target.

Next, to evaluate the contribution of the proposed FiLM-based conditioning strategy, we performed an ablation study using three configurations, as summarized in Table 9. First, the FiLM module was completely removed, resulting in a conventional segmentation network without tissue-specific feature modulation. Second, the FiLM module was retained, but a constant tissue label was assigned to all images, preventing the network from exploiting meaningful tissue-specific information. Finally, the complete proposed model used the correct tissue label to condition the FiLM layers. As shown in Table 9, removing the FiLM module reduced the segmentation performance across all evaluation metrics. Although retaining FiLM with a constant tissue label provided a slight improvement over the model without FiLM, it remained inferior to the proposed model. The proposed model consistently achieved the best performance, demonstrating that both the FiLM module and accurate tissue-specific conditioning contribute to improved segmentation performance.

Furthermore, to investigate the contribution of each component within the proposed GCM, an ablation study was conducted by progressively enabling its constituent components. The evaluated configurations include removal of the GCM; individual atrous convolution branches with dilation rates of 2 and 6; dual atrous branches without self-attention; a self-attention only configuration; and the complete GCM. The quantitative results are summarized in Table 10. Removing the GCM resulted in the largest performance degradation, confirming that contextual feature aggregation is essential for accurate gland segmentation. Employing only a single atrous branch improved performance, with the larger dilation rate of 6 outperforming the smaller dilation rate of 2, indicating that broader contextual information is beneficial for capturing complete gland structures. Combining both atrous branches further improved all evaluation metrics, demonstrating the effectiveness of aggregating fine and coarse contextual information simultaneously.

The self-attention-only configuration also improved segmentation performance compared with the baseline, demonstrating the benefit of modeling long-range spatial dependencies. However, its performance remained lower than that of the dual atrous branch configuration, indicating that global contextual modeling alone cannot fully replace multi-scale feature extraction. Importantly, adding the lightweight self-attention module to the dual atrous branch configuration increased the Dice score from 94.88% to 95.20%, with corresponding improvements in IoU, Precision, Recall, and Conformity. These results demonstrate that the self-attention module contributes complementary contextual information beyond the atrous branches, enabling more accurate feature representation and segmentation. Consequently, the complete GCM achieved the highest performance across all evaluation metrics, confirming the complementary roles of multi-scale atrous convolutions and self-attention in robust gland segmentation.

### 4.6. Comparison with State-of-the-Art Methods

Table 11 presents the per-class segmentation performance of the proposed RCHS Net on the EBHI Seg dataset, while Table 12 provides a quantitative comparison between the proposed RCHS Net and state-of-the-art segmentation methods on the EBHI Seg dataset, including classical encoder–decoder architectures, transformer-based models, and recent lightweight networks. Among the classical CNN-based baselines, U-Net, Seg-Net, and MedT achieve mean Dice scores of 83.45%, 88.40%, and 76.08%, respectively. RCHS-Net surpasses all three by substantial margins, improving over Seg-Net (the strongest classical baseline) by 6.80 percentage points in Dice and 10.77 points in IoU. MedT performs the weakest overall despite its larger model size, further reinforcing that parameter count alone does not determine segmentation quality.

Among transformer-based methods, TransU-Net and Attention U-Net achieve competitive Dice scores of 94.22% and 94.51%, respectively, while SwinU-Net lags notably at 77.36%, suggesting that global attention alone without adequate multi-scale feature recovery is insufficient for fine-grained histopathology segmentation. The proposed RCHS-Net outperforms both TransUNet and Attention U-Net in Dice and IoU while operating at a fraction of their parameter count. CMUNeXt and DMoC-UNet represent strong recent baselines, achieving Dice scores of 94.37% and 94.65%, respectively. Yet, the proposed RCHS-Net surpasses both, demonstrating that the proposed combination of compact multi-scale encoding, the GCM, and FiLM-based class conditioning yields stronger segmentation quality than either convolutional or hybrid transformer designs of comparable scope.

The most relevant comparison is with MAC-Net, the closest prior work in terms of architectural philosophy, which also targets multi-class colorectal segmentation on EBHI-Seg with a lightweight multi-scale attention design. MAC-Net achieves a Dice of 95.08% and IoU of 90.92%, and RCHS-Net improves upon both figures, reaching 95.20% Dice and 91.10% IoU. Notably, RCHS-Net also achieves a higher Recall of 96.27% versus MAC-Net’s 95.65%, indicating fewer missed tissue regions (a clinically significant advantage in cancer screening contexts where false negatives carry greater diagnostic risk than false positives). Furthermore, the proposed RCHS-Net achieves these results with only 243,226 trainable parameters, making it more compact than MAC-Net while maintaining superior performance across all reported metrics. SAM-Path, a foundation-model-based approach, achieves a Dice of 89.12% and IoU of 88.08%, falling below the proposed RCHS-Net on both metrics despite leveraging large-scale pretraining. This result highlights that domain-specific architectural design and targeted training strategies can be more effective than general-purpose foundation models for specialized histopathology segmentation tasks, particularly in data-constrained settings typical of digital pathology.

Overall, the proposed RCHS-Net achieves the highest mean Dice score (95.20%) and IoU (91.10%) among all compared methods, while maintaining competitive Precision (94.74%) and the highest Recall (96.27%). These results confirm that the proposed architecture effectively balances segmentation accuracy, geometric boundary fidelity, and model compactness, establishing a new state of the art for multi-class colorectal histopathology segmentation on the EBHI-Seg benchmark.

For a fair comparison with existing methods, the quantitative results of the competing method are taken directly from their respective source papers. Specifically, the results presented in Table 12 are taken from the MAC-Net paper [26], while those in Table 13 are taken from the Swin-PANet [34] paper. The proposed RCHS-Net was evaluated using the same dataset splits and evaluation metrics reported in the corresponding studies, enabling a direct comparison with the published results.

**Table 11:** Per-class segmentation performance of the proposed RCHS-Net on the EBHI-Seg test set across six colorectal tissue categories, evaluated under oracle class conditioning (ground-truth tissue label provided to the FiLM module at inference). All values are reported as percentages (%).

CRC Stage	Dice Score	IOU	Precision	Recall	Conformity
Adenocarcinoma	93.55	88.30	91.76	96.10	85.40
High-grade IN	94.03	89.13	92.77	95.77	86.43
Low-grade IN	96.66	93.62	96.00	97.44	92.98
Normal	96.23	92.78	97.04	95.48	92.10
Polyp	96.31	92.94	96.23	96.44	92.23
Serrated adenoma	95.28	91.21	94.49	96.22	89.74
Average	95.20	91.10	94.74	96.27	89.44

**Table 12:** Quantitative evaluation of RCHS-Net on the EBHI-Seg benchmark across six colorectal cancer staging categories. Dice score, IoU, Precision, Recall, and Conformity coefficient are reported as percentages (%) (“-” means not reported).

Method	Dice Score	IOU	Precision	Recall
U-Net [6]	83.45	65.50	75.95	77.41
Seg-Net [35]	88.40	80.33	87.10	92.55
MedT [36]	76.08	63.73	78.91	87.18
Attention U-Net [37]	94.51	89.02	87.76	93.65
TransUnet [21]	94.22	89.22	-	-
SAM-Path [38]	89.12	88.08	-	-
SwinUnet [39]	77.36	64.40	-	-
CMUNeXt [40]	94.37	89.72	-	-
DMoC-U-Net [41]	94.65	90.20	-	-
MAC-Net [26]	95.08	90.92	95.83	95.65
RCHS-Net (Proposed)	95.20	91.10	94.74	96.27

To further validate the generalizability of the proposed RCHS-Net beyond the EBHI-Seg benchmark, the model was additionally evaluated on the GlaS dataset, with results presented in Table 13. The GlaS dataset poses a distinct challenge from EBHI-Seg, as it focuses specifically on binary gland segmentation in colorectal histology sections, with significant morphological variability between benign and malignant grades.

As shown in Table 13, the proposed RCHS-Net achieves a Dice score of 93.39% and an IoU of 88.32%, outperforming all competing methods by a considerable margin. Among the baselines, Swin-PANet represents the strongest prior result with a Dice of 91.42% and an IoU of 82.95%, yet the proposed RCHS-Net surpasses it by 1.97 percentage points in Dice and 5.37 points in IoU. UCTransNet, which combines CNN and transformer representations through cross-attention channel fusion, achieves a Dice of 89.84% and IoU of 82.24%, and is likewise outperformed by RCHS-Net on both metrics.

Standard encoder–decoder baselines, including U-Net, U-Net++, and Attention U-Net, cluster between 86 and 87% Dice, while transformer variants such as TransUNet and SwinUNet achieve modest improvements of up to 88.25% Dice. MRUNet, a residual multi-scale variant, achieves 87.72% Dice and 79.39% IoU, falling below RCHS-Net by over 5 and 8 percentage points in Dice and IoU, respectively. The consistent and uniform gap between RCHS-Net and all compared methods on GlaS demonstrates that the performance gains observed on EBHI-Seg are not dataset-specific, but reflect genuine architectural advantages in multi-scale contextual feature extraction and boundary-aware segmentation that transfer across different histopathology benchmarks and task formulations.

**Table 13:** Quantitative comparison of RCHS-Net against state-of-the-art gland segmentation methods on the GlaS benchmark. Dice score and IoU are reported as percentages (%).

Method	Dice Score	IOU
U-Net [6]	86.34	76.81
U-Net++ [42]	87.07	78.10
Attention U-Net [37]	86.98	77.53
MRUNet [43]	87.72	79.39
TransUnet [21]	87.63	79.10
MedT [36]	86.68	77.50
SwinUnet [39]	88.25	79.86
UCTransNet [44]	89.84	82.24
UCTransNet-pre [44]	89.84	82.24
Swin-PANet [34]	91.42	82.95
RCHS-Net (Proposed)	93.39	88.32

Table 14 presents the object-level evaluation of RCHS-Net on the GlaS dataset using the official challenge criteria (object-level F1 score, object-level Dice, and object-level Hausdorff distance, alongside all ten methods from the GlaS challenge leaderboard [32]. RCHS-Net achieves the highest object-level Dice on both Part A (0.912) and Part B (0.794), surpassing all ten challenge submissions, including the top-ranked CUMedVision2. More notably, RCHS-Net achieves the lowest object-level Hausdorff distance on both parts (40.257 on Part A and 112.680 on Part B), indicating superior boundary localization accuracy compared to all challenge methods. On object-level F1, RCHS-Net scores 0.858 on Part A and 0.702 on Part B, ranking fifth and fifth, respectively, behind methods that were developed and optimized specifically for this challenge. These results are particularly significant given that RCHS-Net contains only 243,226 parameters (0.97 MB) and was not purpose-built for the GlaS benchmark but rather designed as a general-purpose lightweight architecture for colorectal histopathology segmentation across multiple tissue categories.

### 4.7. Visualization of Results

**Figure 7:**
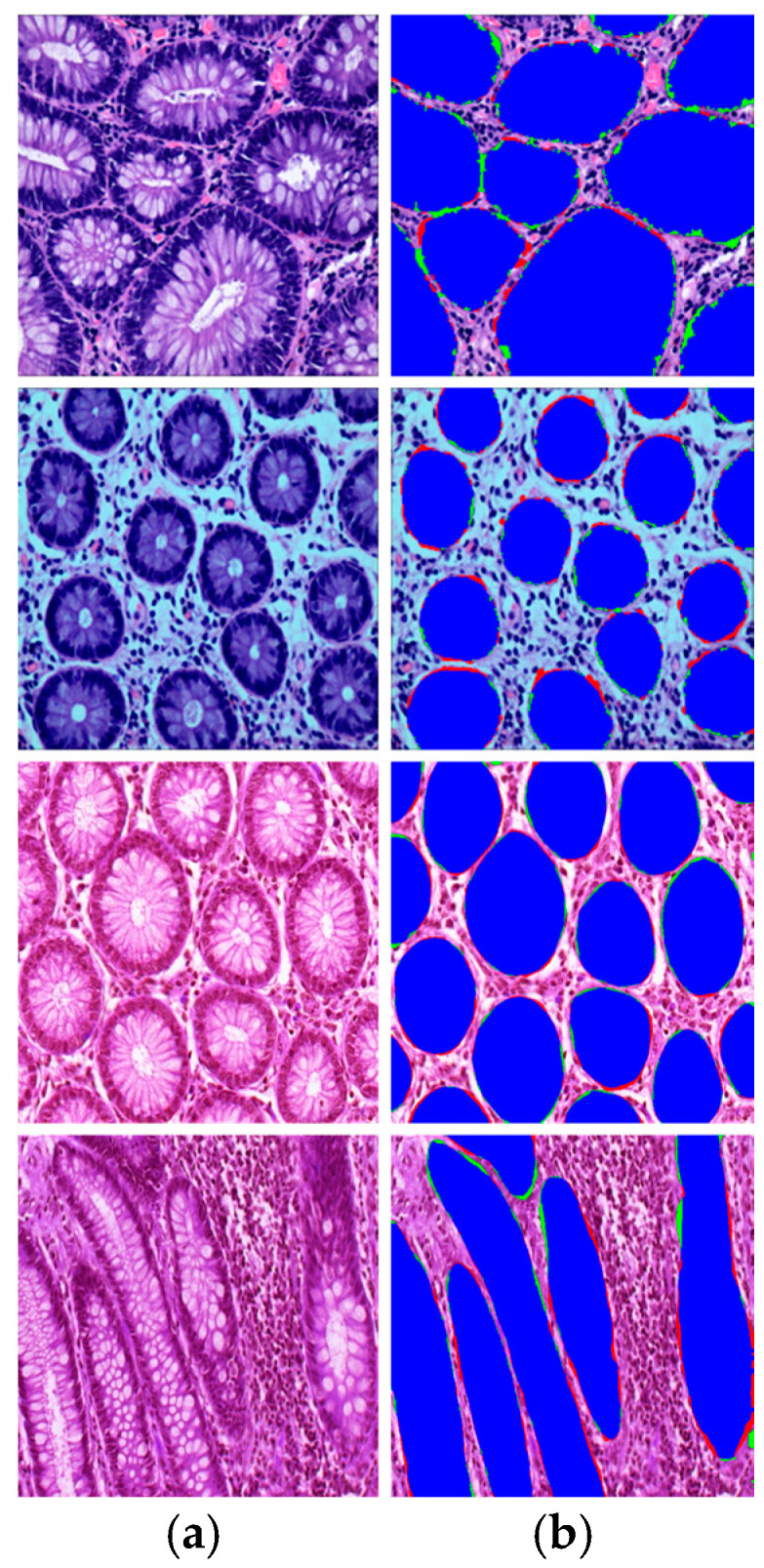
Good segmentation results from RCHS-Net on the EBHI-Seg dataset (top two rows) and GlaS dataset (bottom two rows). (**a**) original H&E-stained patches. (**b**) segmentation overlay where blue = true positives, green = false positives, and red = false negatives.

**Figure 8:**
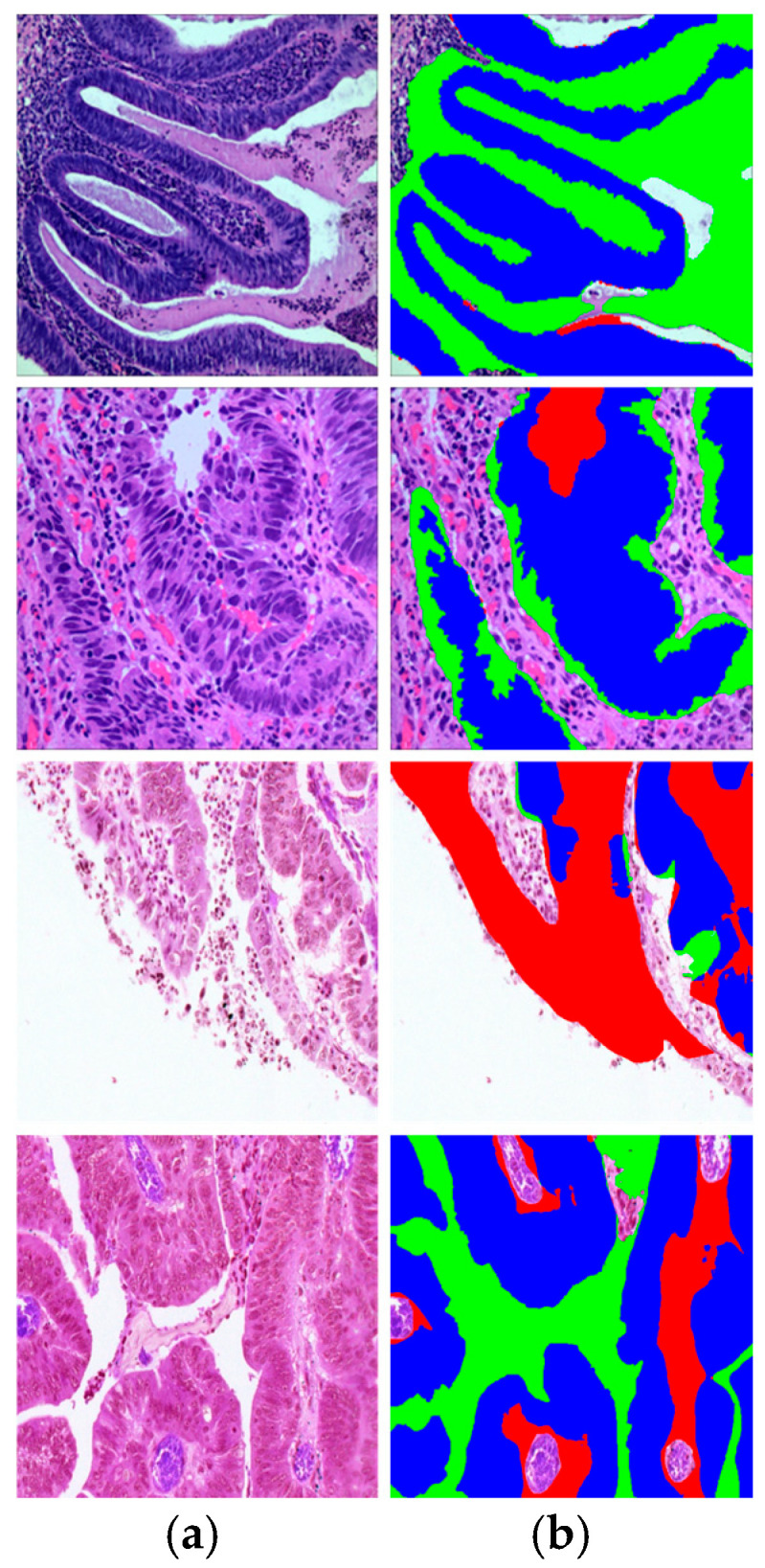
Poor segmentation results from RCHS-Net on the EBHI-Seg dataset (top two rows) and GlaS dataset (bottom two rows). (**a**) original H&E-stained patches. (**b**) segmentation overlay where blue = true positives, green = false positives, and red = false negatives.

To qualitatively assess the segmentation behavior of RCHS-Net beyond aggregate metrics, Figure 7 and Figure 8 present representative good and poor segmentation examples drawn from both the EBHI-Seg and GlaS test sets. In all overlay visualizations, blue regions indicate true positives (correctly segmented tissue), green regions indicate false positives (background incorrectly classified as tissue), and red regions indicate false negatives (tissue regions missed by the model).

Good segmentation examples (Figure 7) demonstrate that RCHS-Net reliably segments well-defined glandular structures across both datasets. In the EBHI-Seg examples (top two rows), the model accurately delineates gland lumens and surrounding epithelial walls, even in tiles containing densely packed crypts at varying sizes, with minimal red or green artifacts visible at the boundaries. The GlaS examples (bottom two rows) similarly show tight boundary adherence along elongated villous structures, confirming that the combination of the GCM’s multi-scale atrous context and the FiLM class conditioning enables the model to handle both compact and elongated glandular morphologies without significant false positives or false negatives. These results are representative of most test samples and are consistent with the high mean Dice (95.20%) and IoU (91.10%) reported on EBHI-Seg.

Poor segmentation examples (Figure 8) reveal three distinct failure modes that account for the residual performance gap. First, in the EBHI-Seg failure cases (top two rows), the model produces fragmented segmentation maps with notable red (false-negative) patches inside larger glandular regions, most commonly in adenocarcinoma tiles where gland walls are structurally disrupted, and the boundary between epithelium and stroma becomes ambiguous. This is a direct consequence of the irregular and disorganized glandular architecture characteristic of high-grade malignancy, where even expert pathologists rely on contextual morphological cues beyond a single 224 × 224 patch. Second, in the GlaS failure cases (bottom two rows), the model generates spurious green (false-positive) regions in areas of loose connective stroma that exhibit staining intensities like glandular epithelium, indicating that stain-domain variation between GlaS scanners introduces foreground–background ambiguity that MixStyle augmentation partially but not fully resolves. Third, across both datasets, small, isolated gland fragments near tile edges tend to be under-segmented, producing localized red artifacts that disproportionately penalize IoU in tiles with high gland boundary-to-area ratios.

**Figure 9:**
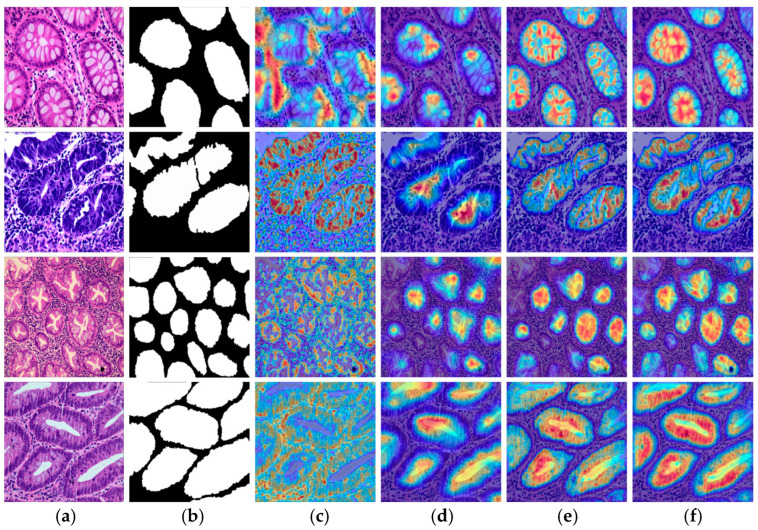
Grad CAM visualization of RCHS-Net for representative test images. From left to right: (**a**) input image, (**b**) ground truth mask, (**c**–**f**) show the Grad CAM activation maps extracted from the encoder, GCM, FPD, and segmentation head, respectively.

To improve the interpretability of the proposed RCHS-Net, gradient-weighted class activation mapping (Grad-CAM) [45] analysis was performed to visualize the image regions that contributed most to the segmentation predictions. Grad-CAM activation maps were extracted from the major components of the network, including the encoder, GCM, FPD, and segmentation head. Representative results are presented in Figure 9. As shown in Figure 9, the encoder stage exhibits broad activation patterns that primarily respond to low-level tissue appearance, including gland textures, epithelial structures, and staining characteristics. After the GCM, the activation maps become increasingly concentrated on complete glandular structures while suppressing surrounding stromal regions, indicating that multi-scale contextual information improves the discrimination of gland morphology. The FPD further refines these responses by enhancing activations along gland boundaries and recovering fine spatial details through multi-scale feature fusion. Finally, the segmentation head produces highly localized activation maps that closely correspond to the final gland regions, demonstrating that the network progressively refines its focus from general tissue characteristics to precise gland localization.

These visualizations suggest that RCHS-Net primarily relies on morphologically relevant glandular regions when generating segmentation predictions, providing additional insight into its internal feature learning process and improving the interpretability of the proposed framework.

### 4.8. Computational Efficiency Analysis

To assess the practical deployability of RCHS-Net in resource-constrained clinical environments, we evaluate its computational profile alongside its segmentation accuracy. Table 15 reports the full set of efficiency metrics measured on a single NVIDIA GPU using a fixed input resolution of 224 × 224, with 100 warm-up iterations followed by 500 timed iterations to ensure stable and reproducible estimates.

RCHS-Net contains only 243,226 trainable parameters (0.97 MB, FP32) and requires 0.35 GFLOPs (176.19 M MACs) per forward pass, which is one to two orders of magnitude lower than transformer-based architectures such as TransU-Net and Swin-U-Net, which typically exceed 20–30 GFLOPs at equivalent input resolution. On GPU (NVIDIA GTX 1070), RCHS-Net achieves a mean inference latency of 8.68 ± 0.79 ms per image (115.21 FPS), with low variance confirming stable, jitter-free throughput. On CPU (Intel Core i7-7700), mean latency is 52.3 ms (19.12 FPS), confirming practical viability in deployments without GPU acceleration. As all experiments are conducted on fixed 224 × 224 patches, WSI-level throughput is not reported, since it would require additional assumptions about the tiling strategy and tissue coverage beyond the experimental scope of this study.

To contextualize the efficiency advantage, Table 15 compares RCHS-Net against MAC-Net [26], which is the strongest accuracy baseline with a published computational-efficiency analysis on the EBHI-Seg dataset. On all hardware-independent measures, RCHS-Net is smaller and computationally cheaper by one to two orders of magnitude: 10.2× fewer parameters, 9.8× smaller model size, and approximately 64× lower GFLOPs. These values confirm that the sub-250K parameter budget and compact architectural design do not constrain segmentation accuracy, but instead reflect a deliberate and effective accuracy–efficiency trade-off. Latency values are included for reference only and are not directly comparable, since MAC-Net’s reported value is averaged over a 20-image batch on a cloud-hosted NVIDIA T4 GPU, whereas ours is single-image latency on a desktop GTX 1070. For the remaining compared methods, source publications do not report efficiency values on EBHI-Seg under comparable conditions, and unverifiable numbers are not included.

### 4.9. Cross-Domain Generalization Evaluation

To evaluate the robustness of the proposed framework under domain shift, cross-dataset experiments were conducted using the EBHI-Seg and GlaS benchmarks. These datasets differ in terms of patient populations, acquisition protocols, staining characteristics, scanner hardware, annotation procedures, and tissue distributions, making them suitable for assessing cross-domain generalization. In the first experiment, RCHS-Net was trained exclusively on the EBHI-Seg training set and directly evaluated on the GlaS test set without any fine-tuning or domain adaptation. In the second experiment, the model was trained on the GlaS training set and evaluated on the EBHI-Seg test set using the same protocol. The quantitative results are summarized in Table 16. As expected, performance decreased under cross-domain evaluation because of the considerable differences between the two datasets. Nevertheless, the proposed framework maintained competitive segmentation accuracy, indicating its ability to generalize across heterogeneous clinical domains. This behavior can be attributed to the combination of multi-scale contextual feature extraction in the GCM and the improved robustness to staining variation provided by photometric augmentation and MixStyle during training.

## 5. Discussion

This study introduced RCHS-Net, a novel resource-efficient encoder–decoder architecture for colorectal histopathology segmentation, demonstrating that state-of-the-art segmentation accuracy can be achieved with fewer than 250K trainable parameters. The experimental findings indicate that carefully designed lightweight architectures can achieve strong segmentation performance while maintaining high computational efficiency, which is particularly critical for deployment within complex, heterogeneous, and resource-constrained healthcare systems. The following findings can be drawn from this study:

- The primary finding of this work is that high-quality colorectal histopathology segmentation does not inherently require large-scale architecture. Previous lightweight approaches on the EBHI-Seg dataset, including MobileViT-U-Net and MAC-Net, demonstrated competitive performance; however, they did not simultaneously achieve a sub-250K parameter footprint, stain domain generalization, and class-conditioned feature adaptation within a unified framework. RCHS-Net addresses all three requirements within a single compact architecture, directly supporting the scalable deployment demands of real-world healthcare systems.
- The ablation analysis demonstrated that the GCM is the most influential component of the proposed framework, contributing to a +1.54% Dice improvement over the baseline encoder and decoder architecture. This finding highlights the importance of multi scale contextual reasoning in colorectal gland segmentation.
- Colorectal glands exhibit substantial morphological variability, ranging from small, isolated crypts in normal tissue to highly disorganized gland clusters in advanced adenocarcinoma. The triple-branch atrous architecture within the GCM effectively addresses this by maintaining complementary receptive fields at dilation rates of r = 2, 6, and 12, corresponding to intra-glandular, individual gland, and gland cluster scales, respectively.
- The lightweight self-attention mechanism integrated within the GCM improves global contextual representation while avoiding the high computational overhead of conventional multi-head self-attention, making it well-suited for resource-constrained edge deployment.
- The FiLM-based class conditioning module achieved an additional +0.73% Dice improvement over the GCM-only configuration, demonstrating the importance of adaptive class-specific feature modulation in class-conditioned binary segmentation. By introducing learnable class-dependent channel scaling and shifting after each encoder stage, FiLM enables class-aware feature representations without increasing backbone complexity. This adaptive modulation is especially relevant in complex healthcare environments where a single model must generalize across diverse tissue presentations.
- The effectiveness of class-conditioned modulation is particularly evident for minority tissue categories such as Serrated Adenoma and Normal tissue, which contain fewer training samples and exhibit markedly different structural characteristics compared with the dominant Adenocarcinoma class, suggesting that FiLM effectively reduces majority-class bias.
- The effectiveness of class-conditioned modulation is particularly evident for minority tissue categories in the EBHI-Seg dataset, such as Serrated Adenoma and Normal tissue, which contain fewer training samples and exhibit markedly different structural characteristics compared with the dominant Adenocarcinoma class. The improved Dice scores obtained for these categories suggest that FiLM effectively reduces the bias of the network toward majority-class distributions.
- MixStyle stain augmentation contributed +0.34% Dice and +0.88% IoU improvement. The larger IoU gain suggests that MixStyle primarily improves boundary consistency and reduces false positives in stain-ambiguous regions, improving robustness across the heterogeneous staining conditions characteristic of real-world multi-site healthcare deployments.

Several limitations of this work should be acknowledged. First, RCHS-Net was evaluated exclusively on H&E-stained histopathology images, and its generalizability to other staining modalities, such as immunohistochemistry, remains unexplored. Second, the model has not been benchmarked on embedded inference hardware such as ARM processors or dedicated neural processing units, and practical deployment latency and energy efficiency remain to be quantified. Third, the current implementation supports only the six predefined EBHI-Seg tissue classes and does not address open-set recognition of unseen pathological categories, which may limit adaptability in evolving clinical environments where novel tissue presentations are encountered.

## 6. Conclusions

In this study, we present RCHS-Net, a novel resource-efficient colorectal histopathology segmentation network tailored for accurate segmentation of H&E-stained tissue across resource-constrained and complex healthcare environments. RCHS-Net integrates a compact multi-scale HistoEncoder with squeeze-and-excitation channel recalibration, a gland context module combining three parallel atrous convolutions (d = 2, d = 6, d = 12) with lightweight self-attention, a feature pyramid decoder for fine-grained spatial reconstruction, and a class-conditioned stain-aware learning strategy combining FiLM modulation with MixStyle augmentation. On the EBHI-Seg benchmark, RCHS-Net achieves a mean Dice of 95.20% and IoU of 91.10%, and on the GlaS benchmark attains a Dice of 93.39% and IoU of 88.32%, surpassing all compared state-of-the-art methods with only 243,226 trainable parameters. These results demonstrate that a single compact model can generalize across diverse tissue categories, staining conditions, and benchmark datasets, directly addressing the adaptive deployment demands of complex real-world healthcare systems.

Despite its strengths, limitations remain: the model has been evaluated on tile-based patch inference rather than whole-slide images, supports only a fixed set of six tissue classes without open-set recognition, and its effectiveness on staining modalities beyond H&E and multi-center datasets requires further investigation. Future work will focus on integrating RCHS-Net with whole-slide image inference pipelines, exploring knowledge distillation to further compress the model below 250K parameters, extending support to additional staining modalities such as immunohistochemistry, and evaluating performance on large-scale multi-center datasets. These directions will further advance the applicability of lightweight AI models for equitable and scalable colorectal cancer screening across diverse global healthcare systems.

## Figures and Tables

**Table 1:** Strengths and weaknesses of the related work.

Type	Method	Strength	Weakness
Handcrafted feature-based	Texture descriptors with SVM and Random Forest classifiers [12]	Benchmark dataset, strong multi-class texture analysis	Limited to classification, no spatial segmentation, limited generalization
	Classical segmentation methods, including k-means, MRF, OTSU, watershed, and the Sobel operator [13]	Simple, interpretable, low computational cost	Low accuracy, poor foreground-background separation, sensitive to staining variations
CNN-Based deep learning methods	Self-configuring framework with automatic adaptation of preprocessing architecture and training [14]	Strong baseline, highly adaptable, state-of-the-art performance	High computational cost, large model size, limited efficiency for real-time use
	PraNet with parallel reverse attention for multi-scale feature aggregation [15]	Accurate polyp boundary detection, effective multi-scale learning	Focused on polyp segmentation, limited generalization to multi-class histology tasks
	Polyp PVT with Pyramid Vision Transformer and cascaded fusion modules [16]	Strong feature representation, effective noise suppression	high computational cost, limited to polyp segmentation tasks
	Enhanced U-Net with multi-scale contextual fusion [17]	Effective multi-scale feature learning, improved segmentation accuracy	Limited global dependency modeling, Potential computational overhead
	Residual encoder, multi-scale fusion, and attention mechanism [18]	Strong multi-scale representation and attention-guided refinement	High computational complexity and limited global context modeling
Vision transformer-based methods	Swin Transformer encoder with a cascaded decoder for segmentation [22]	Strong global context modeling, Effective multi-scale feature learning	High computational and memory cost, Limited fine boundary precision
	Multi-scale token divided and spatial-channel fusion transformer network [23]	Enhanced global feature extraction, robust performance on hyperspectral datasets	High architectural complexity, Limited generalization evidence
	Accurate localization and boundary-aware segmentation, efficient feature extraction [24]	Robust multi-scale feature learning, better generalization potential	High computational complexity, complex architecture, and implementation
Lightweight and resource-efficient architectures	MobileViT as an encoder within the U-Net [25]	Multi-class segmentation capability, High segmentation performance	Limited novelty beyond integration, Limited global context modeling
	Combines multi-scale feature fusion, channel-wise attention, and contextual modeling [26]	Powerful multi-scale feature fusion, Attention-enhanced feature learning	High architectural complexity, Performance drop across domains
	Lightweight U-Net variant inspired by ConvNeXt [25]	Lightweight and efficient design, superior performance with low complexity	Weak global context modeling, potential performance ceiling
	RCHS-Net (Proposed)	Only 243K parameters; dual atrous GCM; FiLM class conditioning; MixStyle stain augmentation	Single-modality (H&E); tile-based inference; not yet evaluated on whole-slide images

**Table 2:** Layer-wise details of the HistoEncoder of the proposed method. H and W denote the input height and width.

Layer	Operation	# of Channels	Stride	Output Spatial Size	Params
Stem	Conv3×3-BN-ReLU6	3 → 24	2	H/2 × W/2	696
Stage 1 (×4 blocks)	ShuffleNet V2 + SE	24 → 32	2 (1st block)	H/4 × W/4	4840
Stage 2 (×5 blocks)	ShuffleNet V2 + SE	32 → 64	2 (1st block)	H/8 × W/8	16,128
Stage 3 (×5 blocks)	ShuffleNet V2 + SE	64 → 128	2 (1st block)	H/16 × W/16	58,880
Total HistoEncoder	—	3 → (32, 64, 128)	—	(H/4, H/8, H/16)	80,544

**Table 3:** Layer-wise details of the GCM of the proposed method. All spatial maps are at H/16 × W/16.

Sub-Module	Operation	In. Channels	Out. Channels/Notes	Params
Compress (PW)	Conv1×1-BN-ReLU6	128	64	8320
Branch r = 2	DW-Sep Conv3×3 (d = 2)	64	64	4800
Branch r = 6	DW-Sep Conv3×3 (d = 6)	64	64	4800
Fuse (PW)	Concat → Conv1×1-BN-ReLU6	128	128	16,640
LightSelfAttn	Q/K (dk = 24), V (128ch) → Attn + 1×1Proj	128	128	39,168
Projection	Conv1×1, BN	128	128	16,640
Residual Add	Projection + Encoder Feature (F_3_)	128	128	0
Total GCM	—	128	128	90,368

**Table 4:** Layer-wise details of the Feature Pyramid Decoder.

Layer	Operation	Input Channels	Output/Notes	Params
Lateral lat3 (PW)	Conv1×1-BN-ReLU6	128 (F3_ctx)	64 at H/16	8320
Lateral lat2 (PW)	Conv1×1-BN-ReLU6	64 (F2)	64 at H/8	4224
Lateral lat1 (PW)	Conv1×1-BN-ReLU6	32 (F1)	64 at H/4	2176
Upsample + Add	Bilinear × 2 + Addition	64	64 at H/8	0
Refinement ref2	DW-Sep Conv3×3-BN-ReLU6	64	64 at H/8 × W/8	4800
Upsample + Add	Bilinear × 2 + Addition	64	64 at H/4	0
Refinement ref1	DW-Sep Conv3×3-BN-ReLU6	64	64 at H/4 × W/4	4800
Output proj (PW)	Conv1×1-BN-ReLU6	64	64	4224
Total FPD	—	32 + 64 + 128	64 at H/4 × W/4	28,544

**Table 5:** Layer-wise details of the prediction heads (SegHead, BoundaryHead) and FiLM class conditioning.

Module	Layer	In. Channels	Out./Notes	Params
SegHead	Conv3×3-BN-ReLU6	64	32	18,497
SegHead	Conv1×1 + Sigmoid + Upsample × 4	32	num_classes (1)	32
BoundaryHead	Conv3×3-BN-ReLU6	64	24	13,872
BoundaryHead	Conv1×1 + Sigmoid + Upsample × 4	24	1 (training only)	25
Class Embedding	Embedding	6 classes	embed_dim = 24	144
FiLM-1	Linear(24, 32 × 2)	embed_dim = 24	Scale+Shift for F1 (32ch)	1600
FiLM-2	Linear(24, 64 × 2)	embed_dim = 24	Scale+Shift for F2 (64ch)	3200
FiLM-3	Linear(24, 128 × 2)	embed_dim = 24	Scale+Shift for F3 (128ch)	6400
Total (Heads + Cond.)	—	—	—	43,770

**Table 6:** Class distribution of the EBHI-Seg dataset.

Category	Images	% of Dataset
Normal	76	3.4%
Polyp	474	21.3%
Low-grade Intraepithelial Neoplasia	639	28.7%
High-grade Intraepithelial Neoplasia	186	8.3%
Serrated Adenoma	58	2.6%
Adenocarcinoma	795	35.7%
Total	2228	100%

**Table 7:** Train, validation, and test split of the EBHI-Seg dataset by category.

Category	Train	Validation	Test
Normal	30	16	30
Polyp	190	94	190
Low-grade Intraepithelial Neoplasia	256	127	256
High-grade Intraepithelial Neoplasia	74	38	74
Serrated Adenoma	23	12	23
Adenocarcinoma	318	159	318

**Table 8:** Ablation study results on the EBHI-Seg dataset. All metrics are mean values across six tissue classes (%). “✓” indicates that the corresponding component is included in the model, while “–” indicates that the component is not included.

Variant/Configuration	SE	GCM	FiLM	BndHead	MixStyle	Dice (%)	IoU (%)	Params
A: Base (HistoEncoder + FPD only)	−	–	–	–	–	90.83	83.47	152K
B: A + SE channel recalibration	✓	–	–	–	–	92.14	85.61	162K
C: B + GCM	✓	✓	–	–	–	93.68	88.07	198K
D: C + FiLM class conditioning	✓	✓	✓	–	–	94.41	89.50	222K
E: D + Auxiliary boundary head	✓	✓	✓	✓	–	94.86	90.22	233K
F: Full RCHS-Net (E + MixStyle augmentation)	✓	✓	✓	✓	✓	95.20	91.10	243K

**Table 9:** Ablation study evaluating the contributions of FiLM conditioning and tissue label information. All values are reported as percentages (%).

Configuration	Dice Score	IOU	Precision	Recall	Conformity
No FiLM	93.93	89.02	92.31	96.21	85.97
FiLM + constant tissue label	94.11	89.18	92.22	96.55	86.94
FiLM + correct tissue label (proposed)	95.20	91.10	94.74	96.27	89.44

**Table 10:** Ablation study evaluating the contributions of the GCM module. All values are reported as percentages (%).

Variant	Dice Score	IOU	Precision	Recall	Conformity
No GCM	91.24	84.50	88.37	95.30	79.07
Atrous r = 2 only	93.95	88.94	92.93	95.45	86.31
Atrous r = 6 only	94.47	89.81	9319	9619	87.77
r = 2 + r = 6 (no Attn)	94.88	90.35	94.08	96.11	88.45
Self-Attention only	93.61	88.41	92.64	95.28	85.95
Full GCM (r = 2 + r = 6 + Attn) (proposed)	95.20	91.10	94.74	96.27	89.44

**Table 14:** Object-level segmentation performance on the GlaS dataset compared against GlaS challenge leaderboard methods [32]. Challenge results are taken from Table 2 of [32].

Method	F1score		Dice_obj_		H_obj_	
	Part A	Part B	Part A	Part B	Part A	Part B
CUMedVision2	0.912	0.716	0.897	0.781	45.418	160.347
ExB1	0.891	0.703	0.897	0.786	57.413	145.575
ExB3	0.896	0.719	0.886	0.765	57.350	145.575
Freiburg2	0.870	0.695	0.876	0.786	57.093	148.463
CUMedVision1	0.868	0.769	0.867	0.800	74.596	153.646
ExB2	0.892	0.686	0.884	0.754	54.785	187.442
Freiburg1	0.834	0.605	0.875	0.783	57.194	146.607
CVML	0.652	0.541	0.644	0.654	155.433	176.244
LIB	0.777	0.306	0.781	0.617	112.706	190.447
vision4GlaS	0.635	0.527	0.737	0.610	107.491	210.105
RCHS-Net (Proposed)	0.858	0.702	0.912	0.794	40.257	112.680

**Table 15:** Hardware-independent efficiency comparison against MAC-Net, the strongest accuracy baseline with a published computational-efficiency analysis on EBHI-Seg.

Metric	RCHS-Net	MAC-Net [26]
Trainable parameters	243,226	2,480,000
Model size (FP32)	0.97 MB	9.46 MB
GFLOPs	0.35 (224 × 224 input)	22.51 (256 × 256 input)
Mean inference latency	8.68 ± 0.79 ms (GTX 1070)	604.8 ms (NVIDIA T4, Colab, batch of 20)
Hardware	Desktop GTX 1070	Cloud T4 (Google Colab)

**Table 16:** Cross-domain evaluation of the proposed RCHS-Net. Dice score, IoU, Precision, and Recall are reported as percentages (%).

Training	Testing	Dice Score	IOU	Precision	Recall
EBHI-Seg	EBHI-Seg	95.20	91.10	94.74	96.27
EBHI-Seg	GlaS	86.72	77.80	87.65	85.94
GlaS	GlaS	93.39	88.32	93.39	88.32
GlaS	EBHI-Seg	88.91	80.42	89.84	88.12

## Data Availability

The datasets analyzed during the current study are publicly available from their respective official repositories. The source code developed for this study is publicly available at https://github.com/tahirlee/RCHS-Net-segmentation-in-histopathology-images (accessed on 18 November 2025). Further details regarding the datasets and implementation are available from the corresponding author upon reasonable request.

## Abbreviations

The following abbreviations are used in this manuscript:

AI	Artificial Intelligence
BN	Batch Normalization
CNN	Convolutional Neural Network
CRC	Colorectal Cancer
DW	Depthwise Convolution
FiLM	Feature-wise Linear Modulation
FP32	Single-Precision Floating-Point (32-bit)
FPD	Feature Pyramid Decoder
GAP	Global Average Pooling
GCM	Gland Context Module
GlaS	Gland Segmentation dataset
GLCM	Gray-Level Co-Occurrence Matrix
GPU	Graphics Processing Unit
H&E	Hematoxylin and Eosin
IoU	Intersection over Union
LBP	Local Binary Patterns
PW	Pointwise Convolution
ReLU	Rectified Linear Unit
RGB	Red, Green, Blue
SAM	Segment Anything Model
SE	Squeeze-and-Excitation
WSI	Whole-Slide Image
